# Functional Outcomes and Reading Speeds following PRESBYOND LBV Using Nonlinear Aspheric Ablation Profiles Combined with Micro-Monovision

**DOI:** 10.1155/2021/2957443

**Published:** 2021-07-31

**Authors:** Sheetal Brar, Smith Snehal Sute, Sheetal N. Bagare, Sri Ganesh

**Affiliations:** Nethradhama Super Speciality Eye Hospital, Bangalore, India

## Abstract

**Purpose:**

To report the functional outcomes and reading speeds following PRESBYOND laser blended vision (LBV) using nonlinear aspheric ablation profiles with micro-monovision with the Carl Zeiss Meditec MEL 90 platform.

**Methods:**

Data have been collected retrospectively for all patients who underwent PRESBYOND LBV using the MEL 90 excimer laser. Postoperative binocular uncorrected distance and near visual acuity, stereo-acuity, contrast sensitivity, and reading performance were compared with pre-op values measured with patient's progressive glasses. Mean follow-up was 6 ± 1.2 months.

**Results:**

Sixty eyes of 30 patients (mean age 50.47 ± 6.43 years) were included. Of these, 18 patients were hyperopic and 12 patients were myopic with mean SE of 1.28 ± 1.38 D and −2.84 ± 1.86 D, respectively. At 6 months, the mean binocular UDVA was ≥−0.03 ± 0.06 log MAR and the mean binocular UNVA was ≥0.22 ± 0.04 log MAR. The uncorrected reading speeds (words per minute) at the preferred reading distance of 46.17 cm, 60 cm, and 80 cm were significantly better (*p* value <0.01), whereas the smallest letter size and reading acuities were comparable to the preoperative values (*p* > 0.05 for all distances). Uncorrected contrast sensitivity log values showed mild reduction; however, this was not statistically significant for any spatial frequency. There was a significant reduction in post uncorrected stereopsis to 89.67 arc sec, compared with pre-op corrected stereopsis (50.67 arc sec); however, it recovered fully with near correction (53.33 arc sec, *p* > 0.05 compared with pre).

**Conclusion:**

PRESBYOND LBV resulted in significantly better reading speeds and satisfactory functional visual outcomes, without a permanent change in stereo-acuity and contrast sensitivity 6 months postoperatively.

## 1. Introduction

The PRESBYOND LBV procedure performed with MEL 90 excimer laser and the CRS-Master successfully combines monovision with extended depth of field achieved by the aspheric laser ablation profile combined with a micro-monovision (−1.50 D) protocol to treat presbyopia. Using this protocol, the intended postoperative refraction is plano for the dominant eye and in the range of −1.00 to −1.50 D for the nondominant eye [1]. Various studies have demonstrated that the procedure was safe and effective across all types of ametropia [1–5].

However, the complete assessment of vision-related abilities should consider visual function (the performance of components of the visual system) and functional vision (visual task-related ability) [6]. Typical visual function tests include assessment of visual acuity, contrast sensitivity, visual fields, tests for binocular vision, colour, depth, and motion perception etc. These properties represent an aspect of visual function, each of which may impact an individual's level of functional vision [7] and thus patient satisfaction after a presbyopia correction surgery.

The goal of functional vision assessment after surgical treatment of presbyopia therefore should be to measure the visual task-related ability under real-world scenarios. Through this study, we aim to evaluate the various visual functions such as distance and near visual acuity, contrast sensitivity, and stereopsis following PRESBYOND LBV. Reading performance, as the visual task-related ability, was also assessed, which has not been described earlier in the context of this procedure.

## 2. Material and Methods

The study was approved by the Institutional Ethics Committee of Nethradhama Eye Hospital and involved retrospective review of electronic medical records of the patients who had undergone PRESBYOND LBV for correction of presbyopia from June 2015 till June 2018. Exclusion criteria were corrected distance visual acuity (CDVA) worse than 20/25 in either eye, previous refractive surgery, corneal and/or lens opacities that may affect vision, optic disc or retinal pathologies, acute or chronic systemic disease, or any kind of immunosuppressive disorder. Only patients whose complete records were available and who had a follow-up of 6 months after surgery were included.

A complete ophthalmic examination was performed for all patients prior to surgery, which included anterior and posterior segment evaluation; dilated refraction; corneal topography with ATLAS topographer (Carl Zeiss Meditec, Jena, Germany) and Pentacam HR (Oculus); and dry eye assessment with Schirmer's I, II, and tear film breakup time (TBUT). Apart from the above, reading performance using Salzburg Reading Desk (SRD), stereo-acuity measurement using the Titmus-C circles (Stereo Optical Co, Chicago, USA), contrast sensitivity using the CSV-1000 chart, and defocus curve testing with defocusing lenses from +2.00 to −3.00 D were also assessed binocularly, with the patients wearing their progressive spectacles.

The reading performance for near and intermediate was evaluated using the Salzburg Reading Desk camera “Version B.5.1.” This device consists of a reading desk with a high-resolution monitor and a laptop where the operating software is displayed. Two infrared video cameras continuously measure the reading distance by stereo photometry. The reading speed and time are recorded with a microphone, incorporated into the SRD monitor. Log-scaled Colenbrander sentences are presented on the monitor in progressively smaller print sizes. A sentence is accepted if it can be read with a minimum speed of 80 words per minute, as this was found to be the minimum threshold for recreational reading in healthy eyes [8, 9].

The reading performance was assessed binocularly before surgery, using patient's own progressive glasses, which were appropriate and improved to the patient's best corrected vision. For near, patients were asked to choose their preferred distance, while for the intermediate, reading performance was evaluated at a fixed distance of 60 cm and 80 cm. Furthermore, the smallest log-scaled print size that could be read effectively (>80 words per minute) was assessed. For near, the letter size ranged from 0.16 to 0.8, while for the intermediate distance, it ranged from 0.16 to 2.0, where 0.16 being the largest and 0.8 and 2.0 being the smallest letter size presented on the monitor of the SRD version evaluated in the current study.

Preoperative refractive workup, verification of the eye dominance, micro-monovision assessment, and anisometropia tolerance were performed as per a standard protocol published earlier [10]. Wavefront aberrometry (WASCA Analyzer; Carl Zeiss Meditec, Jena, Germany) was used to measure the ocular wavefront aberrations in scotopic condition, and data at a diameter of 6 mm were analyzed.

All surgical procedures were performed by two experienced refractive surgeons (SG and SB) using the VisuMax femtosecond laser and MEL 90 excimer laser (both Carl Zeiss Meditec, Jena, Germany). The CRS-Master software platform (both Carl Zeiss Meditec) was used to design the aspheric ablation profile using the ocular wavefront data obtained by the WASCA aberrometer, which was then exported for treatment with the MEL 90 excimer laser. The surgical procedure was similar to that of a standard femtosecond LASIK treatment. Flaps were created with the VisuMax femtosecond laser using a 100–120 *μ*m flap thickness. Stromal aspheric ablation was performed using the MEL 90 excimer laser with a 6.45 ± 0.19 (Range: 6.00–6.80) mm optical zone and 2.2 mm transition zone.

Postoperatively, patients were followed up at day 1, 2 weeks, 3 months, and 6 months. On all follow-up visits, measurement of uncorrected distance and near visual acuity (UDVA and UNVA), CDVA, manifest refraction, and a patient questionnaire regarding their satisfaction following the procedure were obtained. On all visits from 2 weeks onwards, reading performance, stereo-acuity, contrast sensitivity, and defocus curve testing were repeated binocularly without correction to evaluate functional outcomes.

Statistical analysis was performed using the SPSS statistical package (version 17.0; SPSS, Inc., Chicago, IL). Data were checked for normality before subjecting to analysis. If the data were normally distributed, paired Student's *t*-tests were performed to compare the mean values of UDVA, UNVA, CDVA, UIVA, contrast sensitivity, stereo-acuity, and reading performance-related parameters. If the data distribution was not normal, the Wilcoxon signed-rank test was used. A *p* value less than 0.05 was considered statistically significant.

## 3. Results

A total of 30 patients with mean age of 50.47 ± 6.43 years (range 41–64 years), who underwent bilateral treatment with PRESBYOND LBV for myopia (*n* = 12 patients) or hyperopia (*n* = 18 patients), with or without astigmatism were included in the study.  Table 1 shows the preoperative demographic details of all the patients included in the study. The mean preoperative manifest spherical equivalent (SE) of the dominant eyes was −0.30 ± 2.45 D (range: −7.25 to +4.00 D), and that of the nondominant eyes was −0.47 ± 2.70 D (range: −5.75 to +3.625 D). Mean follow-up was 6.00 ± 1.2 months.

### 3.1. Visual and Refractive Outcomes

**Figure 1:**
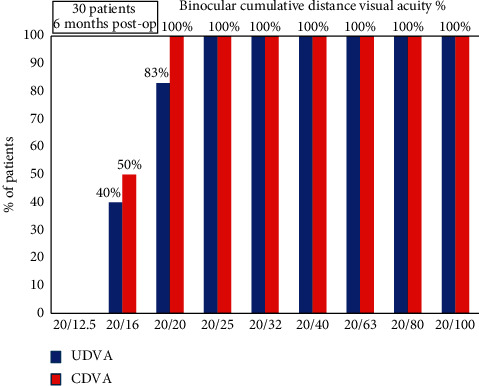
Cumulative histogram for binocular UDVA and CDVA at 6 months post-op.

Eighty-three percent (*n* = 25) patients achieved a binocular cumulative uncorrected distance visual acuity of 20/20 or better, while all patients had a binocular UDVA of 20/25 or better (Figure 1).

**Figure 2:**
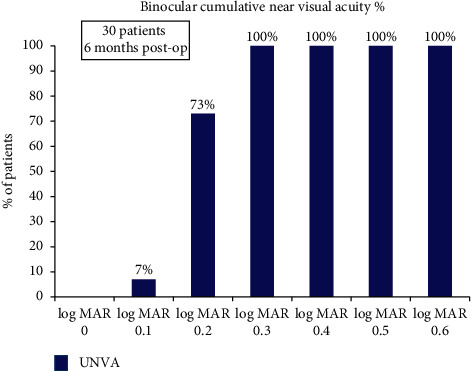
Cumulative histogram for binocular UNVA at 6 months post-op.

Additionally, all patients achieved binocular cumulative uncorrected near visual acuity of 0.3 log MAR, and 73% patients could read 0.2 log MAR or better binocularly (Figure 2).

The mean post-op SE refraction of the dominant eyes was −0.03 ± 0.29 D (range: −0.5 to +0.62 D) and that of the nondominant eyes was −1.26 ± 0.40 D (range: −2.25 to −0.75 D), Table 2.

**Figure 3:**
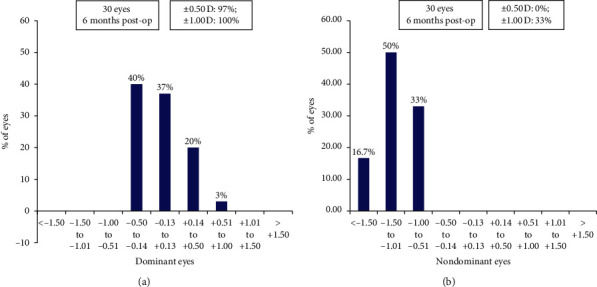
Histogram showing the accuracy to the intended spherical equivalent refraction for (a) dominant and (b) nondominant eyes at 6 months post-op.

Ninety-seven (*n* = 29) percent dominant eyes were within ±0.50 D, and all eyes were within ±1.00 D of SE predictability. Of the nondominant eyes, 66.7% (*n* = 20) were within −1.00 to −1.50 D of SE predictability (range −0.75 to −2.25 D) (Figure 3).

**Figure 4:**
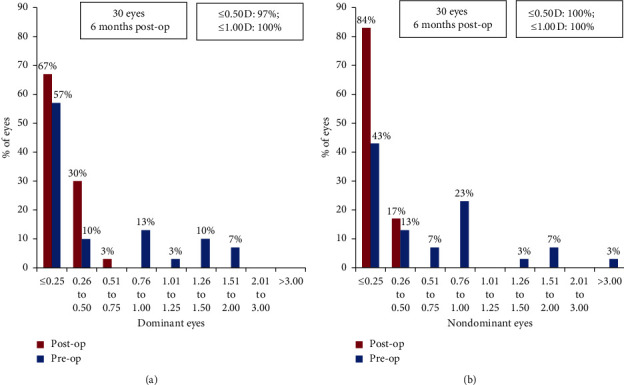
Histogram showing change in refractive astigmatism for (a) dominant and (b) nondominant eyes at 6 months postoperatively.

Ninety-seven (*n* = 29) percent of dominant eyes and 100% of nondominant eyes were within a refractive astigmatism of ±0.5 D (Figure 4).

### 3.2. Reading Performance

The preferred reading distance increased from 41.8 ± 4.87 cm pre-op to 46.16 ± 5.40 cm postsurgery, which was statistically significant (*p*=0.01).

**Figure 5:**
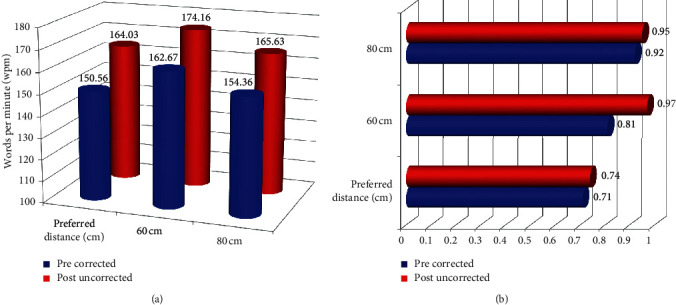
Binocular reading performance evaluated using SRD at preferred reading distance, 60 cm, and 80 cm. (a) Reading speed. (b) Letter size.

The reading speeds at the near preferred distance, 60 cm, and 80 cm showed significant improvement, compared with the pre-op values recorded with patient's progressive glasses. The reading speed at 60 cm was significantly better than the reading speed at 80 cm (Table 2, Figure 5).

There was, however, no significant difference for the post-op uncorrected reading acuity and smallest letter read (with a minimum reading speed of 80 wpm) at the preferred reading distance, 60 cm, and 80 cm versus their pre-op corrected values (Table 2, Figure 5).

### 3.3. Stereopsis

The mean postoperative binocular uncorrected stereopsis (89.67 ± 35.95 arc sec) was significantly lower than the preoperative corrected value of 50.67 ± 17.20 arc sec (*p*=0.01). However, with near correction, the stereo-acuity improved to 53.33 ± 16.25 arc sec, which was comparable with the preoperative values (*p*=0.53).

### 3.4. Subgroup Analysis

Subgroup analysis was done between two groups for patients aged “less than 55 years” and “55 years and above,” with regard to binocular visual outcomes, reading performance in terms of reading acuity, letter size and reading speed, and stereopsis. No significant change was found between groups for any of the analyzed parameter (Table 3).

### 3.5. Contrast Sensitivity

**Figure 6:**
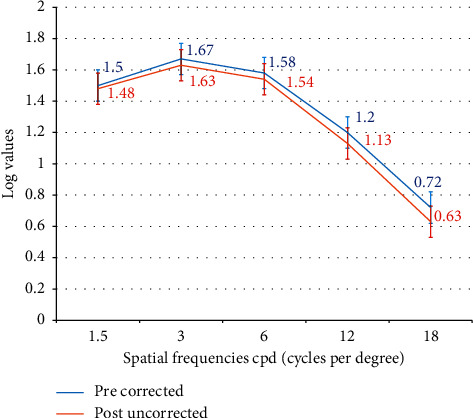
Contrast sensitivity (F.A.C.T) at 6 months post-op.

A mild drop in the contrast sensitivity was observed at 6 months for post uncorrected log values at all spatial frequencies, which was not significantly different from the pre-op corrected values (*p* value >0.05 for all spatial frequencies) (Figure 6).

### 3.6. Defocus Curve

**Figure 7:**
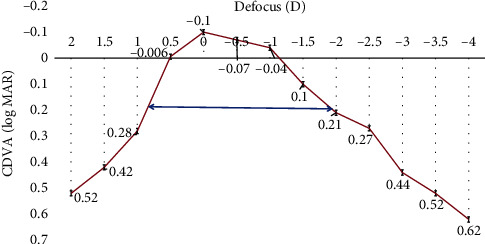
Defocus curve (distance corrected) at 6 months post-op.

Binocular defocus curves were plotted with distance correction from +2.00 to −4.00 D. The curve showed a single peak at 0.00 D, corresponding to a visual acuity of −0.1 log MAR, followed by a gradual decline. A mean visual acuity of 0 log MAR or better was observed within the defocus range of +0.50 to −1.00 D, and a full range of functional vision, i.e., 0.2 log MAR(20/30) or better was achieved from +0.50 to −2.00 D of defocus (Figure 7).

### 3.7. Safety and Complications

**Figure 8:**
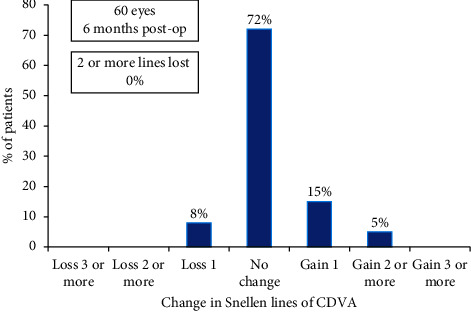
Histogram showing the change in Snellen lines of corrected distance visual acuity (CDVA).

Twenty percent (12) eyes gained one or more lines, 8% (5) eyes lost one line, while 72% (43) eyes did not show any change in CDVA at 6 months post-op (Figure 8).

The loss in one line of CDVA in 5 eyes could be explained by higher induced aberrations in hyperopic eyes, post-op LASIK-induced dry eye, loss in contrast, etc. This was, however, not clinically significant as binocular evaluation showed good outcomes for distance vision.

None of the eyes had any short- or long-term complications such as diffuse lamellar keratitis, infection, flap wrinkles, dislocation, and epithelial ingrowth. No eye in this cohort required enhancement for distance or near vision at the end of 6-month follow-up.

### 3.8. Patient Satisfaction Scores

Table 4 shows the subjective questionnaire used to assess patient satisfaction following PRESBYOND LBV at 6 months. The mean satisfaction scores for distance, intermediate and near were 97.97 ± 2.13, 99.36 ± 0.64 and 96.84 ± 2.36 respectively. Twenty-eight (93.3%) patients were satisfied for distance, while 26 (86.6%) patients were satisfied for near vision. All patients had 100% satisfaction for intermediate vision related activities. No patient complained of severe glare or haloes. However, two (6.6%) patients reported grade 1 (mild) dysphotopsia at the end of 6 months follow-up.

## 4. Discussion

As earlier described, laser blended vision (LBV) involves a combination of controlled induced corneal spherical aberrations and a micro-monovision protocol, aiming at a micro-monovision targeting mild myopia of −1.50 D or less for the near eye, irrespective of the age [10]. In addition, the optimized aspheric ablation profile is intended to increase the depth of field of each eye, resulting in creation of a blend zone to enable continuous distance to intermediate to near vision between the two eyes. Due to the above factors, PRESBYOND LBV appears to be advantageous over traditional LASIK monovision, which was found to be associated with side effects such as poor intermediate vision, reduced contrast sensitivity, loss of stereopsis, and increased photic phenomena and longer adaptation time; all factors potentially reducing patient satisfaction [11, 12]. The main aim of this study was to evaluate the functional aspects of vision and reading speeds following PRESBYOND LBV, of which data are limited in the literature.

Castro et al. simulated the anisocoria generated by corneal inlays using a small aperture contact lens and demonstrated a significant deterioration of stereo-acuity for near and intermediate distances [13]. Also, studies with LASIK monovision demonstrated that in a proportion of these patients, stereo-acuity is lost and that once lost, it does not recover [11, 14, 15]. Our results were similar to those of Reinstein et al., who found that although postoperative uncorrected stereo-acuity was lower than preoperative near-corrected stereo-acuity after LBV, a functional level of stereo-acuity was maintained postoperatively; 68% of patients had stereo-acuity of 100 sec or better and 93% had stereo-acuity of 200 sec or better [16].

In our series, all patients had stereo-acuity of 140 sec or better, while 70% (21) patients had stereo-acuity of 60 sec or better. Near-correction restored preoperative near-corrected stereo-acuity in all the patients, suggesting that PRESBYOND LBV did not lead to irreversible loss of stereo-acuity.

Uthoff et al. investigated the outcomes of simultaneous correction of presbyopia and ametropia by a PresbyMAX (bi-aspheric cornea modulation) technique, based on the creation of a central hyper positive area for near vision and leaving the pericentral cornea for far vision [17]. In a series of 60 eyes of 30 patients, they reported up to 13% eyes losing 2 lines of CDVA. On the other hand, our study involving the similar number of eyes showed better safety, as only 8% eyes lost 1 line and no eye lost more than 2 lines of CDVA. This may suggest that procedures based on creating corneal multifocality for presbyopia treatment may result in drop in distance visual acuity, which is more than PRESBYOND LBV, probably due to higher induced aberrations and worsening of contrast sensitivity.

Studies evaluating the results of peripheral PresbyLASIK algorithm [18] and hybrid techniques [15] (based on targeting a postsurgical corneal asphericity) reported a reduction in postoperative contrast sensitivity for all spatial frequencies. However, Zhang et al. who evaluated contrast sensitivity following PRESBYOND LBV found that, compared in logarithmic scale, the change in binocular contrast sensitivity from the preoperative values in mesopic and photopic conditions was not significantly different at any frequency. The change of AULCSF was not significant either, changing from 1.38 to 1.41 and 1.42 to 1.43 in mesopic and photopic conditions [19]. Our results were similar to those of Zhang et al. wherein we did not observe any significant difference between the pre-op corrected versus post-op uncorrected contrast sensitivity values at any spatial frequency at 6 months.

Charman [20] suggested that, extended binocular depth of focus for presbyopia treatments can be achieved by aiming for residual higher-order aberrations (HOAs). Although, in the present study, we did not analyse change in aberrations, in a previously published paper, we calculated the same based on the experiments performed by Yi et al. [21] according to which the theoretical depth of focus achieved was up to 1.55 D in hyperopic and 0.48 D in the myopic eyes. This could be reflected in the defocus curve, which was charted +2.00 to −4.00 D. It could be inferred that PRESBYOND LBV resulted in a functional vision [22] of 0.2 log MAR (20/32) or better from +0.50 to −2.00 D, suggesting a theoretical depth of defocus of 2.50 D.

Reading is one of the most vital and common skills for engaging, communicating and interpreting ideas. Any visual loss that affects reading ability will have a disproportionate impact on a patient's quality of life. Reading speed more closely aligns with task performance than visual acuity metrics [23]. In this study, reading performance at near was evaluated at the patients preferred distance, as it was suggested that reading distance could vary considerably depending upon the posture, body size, habits, illumination, type of spectacles, and other factors [9]. However, for intermediate, fixed distances of 60 and 80 cm were selected, as the “blend zone” created in the this range following PRESBYOND LBV, is supposed to provide a continuous vision from distance through near. It was found that the reading speed at 60 cm was significantly better than 80 cm, reflecting the expected outcome of the treatment planning, wherein the average monovision target achieved at 6 months, was −1.26 D.

Reading speeds using the Salzburg reading desk have been evaluated earlier for various presbyopia-correcting modalities. Dexl et al. assessed reading performance following a small aperture corneal inlay at 2 years follow-up using SRD [24]. In their study, the mean uncorrected post-op reading speed was 146 ± 20 wpm, which was lower than the pre-op value of 153 ± 23 wpm, measured with reading addition at patient's preferred distance (39.5 cm). Another study evaluating the reading performance following Tecnis Symfony extended depth of focus IOL found that the average post-op binocular reading speed at patient's preferred distance (41 cm) was 109 ± 33 wpm [25]. This appears to be markedly low compared with the reading speed achieved after PRESBYOND LBV in the present study, where in the post-op reading speed (164 ± 18 wpm) was significantly better than the pre-op value of 150 ± 7.29 wpm, measured with patient's progressive spectacles at a preferred distance chosen by the patient (46.16 cm). This may suggest that PRESBYOND LBV may result in better reading performance than procedures aiming at extending the depth of focus, achieved through means other than controlled induction of spherical aberrations, as utilized in PRESBYOND. This may, however, need further data for verification.

The significant improvement in post-op reading speeds seen in our study may be attributed to various factors. First and foremost, pre-op measurements were performed with patients own progressive spectacles, which may cause visual acuity drop-off, image distortion, and constant need to adapt their gaze and head movements to each lens design, causing patient discomfort [26]. These phenomena obviously improved after PRESBYOND LBV, which additionally also widened the field of vision, making reading a more comfortable task. Second, it could be due to the learning effect of repeating the test sentences by the patients [27].

In conclusion, PRESBYOND LBV effectively demonstrated improved visual and refractive results as well as functional outcomes at 6 months. The procedure delivered a wide range of functional vision, without any permanent change in contrast sensitivity or stereopsis, compared with the previously published presbyopia-correcting procedures. Reading speeds evaluated under standardized conditions were significantly better after surgery than patient's progressive glasses, indicating both subjective and objective improvement in everyday reading ability. To the best of our knowledge, this is the first study evaluating reading performance following PRESBYOND LBV. We believe this further enhances our understanding about the functional outcomes and reading ability after PRESBYOND LBV and how it affects the quality of life and patient satisfaction postoperatively.

## Figures and Tables

**Table 1:** Patient demographics and preoperative data.

*Patient demographics*		
Total no. of eyes	60	
Total no. of patients	30	
Male: female	14 : 16	
Age (years)	50.47 ± 6.43	
Binocular UDVA (log MAR)	0.48 ± 0.39	
Binocular CDVA (log MAR)	−0.01 ± 0.06	
Binocular DCNVA (log MAR)	0.23 ± 0.06	
K mean (D)	43.89 ± 1.38	
CCT (*μ*)	529.93 ± 34.10	
Optical zone (mm)	6.45 ± 0.19	
Flap thickness (*μ*)	118.33 ± 8.89	
Ablation depth (*μ*)	49.3 ± 26.57	
Post-op RST (*μ*)	356.30 ± 43.97	
*Z* (4, 0)	−0.23 ± 0.28	

*Visual acuity and refraction*		
Parameter (mean ± SD)	Dominant eyes (*n* = 30)	Nondominant eyes (*n* = 30)

Sphere (D)	−0.075 ± 1.72	−0.25 ± 2.40
Cylinder (D)	−0.24 ± 0.80	−0.41 ± 0.91
SE (D)	−0.19 ± 1.93	−0.47 ± 2.70
UDVA (log MAR)	0.48 ± 0.39	0.62 ± 0.41
CDVA (log MAR)	−0.01 ± 0.06	−0.01 ± 0.08
DCNVA (log MAR)	0.23 ± 0.06	0.22 ± 0.05

D: dioptre, SE = spherical equivalent, UDVA = uncorrected distance visual acuity, CDVA = corrected distance visual acuity, DCNVA = distance corrected near visual acuity, K = keratometry, CCT = central corneal thickness, and RST = residual stromal thickness.

**Table 2:** Visual acuity outcomes and reading performance at 6 months postoperatively.

Parameter	Dominant eyes (*n* = 30)	Nondominant eyes (*n* = 30)	

Sphere (D)	0.02 ± 0.23	−1.20 ± 0.36	
Cylinder (D)	−0.09 ± 0.31	−0.11 ± 0.19	
SE (D)	−0.03 ± 0.29	−1.26 ± 0.40	
UDVA (log MAR)	−0.03 ± 0.67	0.39 ± 0.19	
CDVA (log MAR)	−0.04 ± 0.54	−0.03 ± 0.05	

*Binocular visual acuity (log MAR)*			
Parameter	Uncorrected	Distance corrected	*p* value
Distance	−0.032 ± 0.06	−0.06 ± 0.05	0.06
Near	0.22 ± 0.04	0.4 ± 0.11	0.01

*Reading performance*			
Reading acuity (log MAR) (mean ± SD)	Pre corrected	Post uncorrected	*p* value
40 cm	0.043 ± 0.12	0.031 ± 0.11	0.70
60 cm	0.049 ± 0.17	0.046 ± 0.06	0.92
80 cm	0.117 ± 0.04	0.101 ± 0.05	0.21

Letter size (log scale) (mean ± SD)			
40 cm	0.71 ± 0.12	0.74 ± 0.08	0.36
60 cm	0.91 ± 0.21	0.97 ± 0.16	0.19
80 cm	0.92 ± 0.17	0.95 ± 0.26	0.60

Reading speed (WPM) (mean ± SD)			
40 cm	150.56 ± 7.3	164.03 ± 18.62	0.01
60 cm	162.67 ± 6.38	174.16 ± 9.55	0.01
80 cm	154.36 ± 7.29	165.63 ± 18.06	0.01

Reading performance at intermediate distance (60 cm versus 80 cm) at 6 months post-op			
Reading acuity (log MAR)	0.52		
Letter size	0.16		
Reading speeds (WPM)	0.02		

**Table 3:** Subgroup analysis for patients aged “less than 55 years” and “55 years and above.”

Parameter (mean ± SD)	Less than 55	55 and above	*p* value

*Binocular visual acuity (log MAR)*			
Distance uncorrected	−0.037 ± 0.06	−0.01 ± 0.07	0.46
Distance corrected	−0.06 ± 0.04	−0.04 ± 0.05	0.39
Near uncorrected	0.22 ± 0.03	0.24 ± 0.05	0.27
Near corrected	0.38 ± 0.10	0.45 ± 0.15	0.16

*Reading performance*			
Reading acuity (log MAR)	Less than 55	55 and above	*p* value
40 cm (pre corrected)	0.03 ± 0.11	0.06 ± 0.14	0.6
(Post uncorrected)	0.02 ± 0.11	0.04 ± 0.11	0.64
60 cm (pre corrected)	0.05 ± 0.15	0.04 ± 0.23	0.86
(Post uncorrected)	0.04 ± 0.06	0.04 ± 0.05	0.76
80 cm (pre corrected)	0.11 ± 0.05	0.12 ± 0.01	0.71
(Post uncorrected)	0.1 ± 0.05	0.10 ± 0.04	0.82

*Letter size (log scale)*			
40 cm (pre corrected)	0.71 ± 0.12	0.72 ± 0.11	0.97
(Post uncorrected)	0.74 ± 0.08	0.73 ± 0.08	0.77
60 cm (pre corrected)	0.11 ± 0.03	0.12 ± 0.03	0.82
(Post uncorrected)	0.97 ± 0.17	0.98 ± 0.14	0.92
80 cm (pre corrected)	0.94 ± 0.16	0.86 ± 0.20	0.28
(Post uncorrected)	0.96 ± 0.29	0.93 ± 0.20	0.96

*Reading speed (WPM)*			
40 cm (pre corrected)	150.45 ± 7.28	150.87 ± 7.82	0.89
(Post uncorrected)	165.09 ± 20.02	161.12 ± 14.85	0.61
60 cm (pre corrected)	162.72 ± 6.51	162.5 ± 6.41	0.93
(Post uncorrected)	173.90 ± 9.99	174.87 ± 8.80	0.81
80 cm (pre corrected)	154.04 ± 7.39	155.25 ± 7.42	0.69
(Post uncorrected)	166.63 ± 17.91	162.87 ± 19.44	0.62

*Stereopsis*			
Pre-op uncorrected	50 ± 17.18	52.5 ± 18.32	0.73
Post-op uncorrected	82.27 ± 36.40	96.25 ± 36.22	0.55
Post-op uncorrected	52.27 ± 16.59	56.25 ± 15.97	0.56

**Table 4:** Postoperative patient satisfaction score and dysphotopsia grading.

*Patient satisfaction score (mean ± SD)*	
Distance vision	97.97 ± 2.13
Intermediate vision	99.36 ± 0.64
Near vision	96.84 ± 2.36

*Postoperative spectacle independence [% of patients] (n* *=* *30)*	
Distance vision	93.33% (28)
Intermediate vision	100% (30)
Near vision	86.67% (26)

*Postoperative dysphotopsia grading [% of patients] (n* *=* *30)*	
Grade 0 (nil)	93.33% (28)
Grade 1 (mild)	6.66% (2)
Grade 2 (moderate)	0% (0)
Grade 3 (severe)	0% (0)

Satisfaction questionnaire included scores ranging from 0 to 100, where 0 indicated not at all satisfied and 100 indicated completely satisfied, without the need of spectacles. Postoperative spectacle independence was evaluated as the percentage of patients who were completely free or did not feel the need of glasses for a particular distance. Dysphotopsia grading was done as per the following questionnaire: 0 = nil, no dysphotopsia symptoms experienced; 1 = mild, minimal dysphotopsia not affecting night vision and routine activities; 2 = moderate, dysphotopsia symptoms affecting night vision and routine activities, but manageable; 3 = severe, bothersome dysphotopsia, severe enough to interfere in routine activities.

## Data Availability

The data can be made available on request from Dr Sandhya, Institutional Ethics Committee in-charge of Nethradhama Superspeciality Eye Hospital, Bangalore, who can be contacted at sandhyakrish@gmail.com.

## References

[1] Reinstein D. Z., Archer T. J., Gobbe M. (2011). LASIK for myopic astigmatism and presbyopia using non-linear aspheric micro-monovision with the Carl Zeiss Meditec MEL 80 platform. *Journal of Refractive Surgery*.

[2] Reinstein D. Z., Couch D. G., Archer T. J. (2009). LASIK for hyperopic astigmatism and presbyopia using micro-monovision with the Carl Zeiss Meditec MEL80 platform. *Journal of Refractive Surgery*.

[3] Reinstein D. Z., Carp G. I., Archer T. J., Gobbe M. (2012). LASIK for presbyopia correction in emmetropic patients using aspheric ablation profiles and a micro-monovision protocol with the Carl Zeiss Meditec MEL 80 and VisuMax. *Journal of Refractive Surgery*.

[4] Gifford P., Kang P., Swarbrick H., Versace P. (2014). Changes to corneal aberrations and vision after Presbylasik refractive surgery using the MEL 80 platform. *Journal of Refractive Surgery*.

[5] Falcon C., Norero Martínez M., Sancho Miralles Y. (2015). Laser blended vision (vision combinée) pour la correction de la presbytie: résultats à 3ans. *Journal Français d’Ophtalmologie*.

[6] Bennett C. R., Bex P. J., Bauer C. M., Merabet L. B. (2019). The assessment of visual function and functional vision. *Seminars in Pediatric Neurology*.

[7] Lennie P., Van Hemel S. B., National Research Council (US) (2002). Committee on disability determination for individuals with visual impairments. *Visual Impairments: Determining Eligibility for Social Security Benefits*.

[8] Hirnschall N., Motaabbed J. K., Dexl A., Grabner G., Findl O. (2014). Evaluation of an electronic reading desk to measure reading acuity in pseudophakic patients. *Journal of Cataract and Refractive Surgery*.

[9] Dexl A. K., Schlögel H., Wolfbauer M., Grabner G. (2010). Device for improving quantification of reading acuity and reading speed. *Journal of Refractive Surgery*.

[10] Ganesh S., Brar S., Gautam M., Sriprakash K. (2020). Visual and refractive outcomes following laser blended vision using non-linear aspheric micro-monovision. *Journal of Refractive Surgery*.

[11] Garcia-Gonzalez M., Teus M. A., Hernandez-Verdejo J. L. (2010). Visual outcomes of LASIK-induced monovision in myopic patients with presbyopia. *American Journal of Ophthalmology*.

[12] Fawcett S. L., Herman W. K., Alfieri C. D., Castleberry K. A., Parks M. M., Birch E. E. (2001). Stereoacuity and foveal fusion in adults with long-standing surgical monovision. *Journal of American Association for Pediatric Ophthalmology and Strabismus*.

[13] Castro J. J., Ortiz C., Jiménez J. R., Ortiz-Peregrina S., Casares-López M. (2018). Stereopsis simulating small-aperture corneal inlay and monovision conditions. *Journal of Refractive Surgery*.

[14] Verdoorn C. (2017). Comparison of a hydrogel corneal inlay and monovision laser in situ keratomileusis in presbyopic patients: focus on visual performance and optical quality. *Clinical Ophthalmology*.

[15] Alarcón A., Anera R. G., Villa C., del Barco L. J., Gutierrez R. (2011). Visual quality after monovision correction by laser in situ keratomileusis in presbyopic patients. *Journal of Cataract and Refractive Surgery*.

[16] Reinstein D. Z., Archer T. J., Carp G. I. Non-linear aspheric ablation profile for presbyopic corneal treatment using with the MEL 80/90 and CRS master. Pesbyond module.

[17] Uthoff D., Pölzl M., Hepper D., Holland D. (2012). A new method of cornea modulation with excimer laser for simultaneous correction of presbyopia and ametropia. *Graefe’s Archive for Clinical and Experimental Ophthalmology*.

[18] Pinelli R., Ortiz D., Simonetto A., Bacchi C., Sala E., Alió J. L. (2008). Correction of presbyopia in hyperopia with a center-distance, paracentral-near technique using the technolas 217z platform. *Journal of Refractive Surgery*.

[19] Zhang T., Sun Y., Weng S. (2016). Aspheric micro-monovision LASIK in correction of presbyopia and myopic astigmatism: early clinical outcomes in a Chinese population. *Journal of Refractive Surgery*.

[20] Charman W. N. (2004). Ablation design in relation to spatial frequency, depth-of-focus, and age. *Journal of Refractive Surgery*.

[21] Yi F., Robert Iskander D., Collins M. (2011). Depth of focus and visual acuity with primary and secondary spherical aberration. *Vision Research*.

[22] Uy E., Go R. (2009). Pseudoaccommodative cornea treatment using the NIDEK EC-5000 CXIII excimer laser in myopic and hyperopic presbyopes. *Journal of Refractive Surgery*.

[23] Wolffsohn J. S., Davies L. N. (2019). Presbyopia: effectiveness of correction strategies. *Progress in Retinal and Eye Research*.

[24] Dexl A. K., Seyeddain O., Riha W. (2012). Reading performance and patient satisfaction after corneal inlay implantation for presbyopia correction: two-year follow-up. *Journal of Cataract and Refractive Surgery*.

[25] Attia M. S. A., Auffarth G. U., Kretz F. T. A. (2017). Clinical evaluation of an extended depth of focus intraocular lens with the Salzburg reading desk. *Journal of Refractive Surgery*.

[26] Forkel J., Reiniger J. L., Muschielok A., Welk A., Seidemann A., Baumbach P. (2017). Personalized progressive addition lenses: correlation between performance and design. *Optometry and Vision Science*.

[27] Arad T., Baumeister M., Bühren J., Kohnen T. (2017). Evaluation of a device for standardized measurements of reading performance in a prepresbyopic population. *European Journal of Ophthalmology*.

